# “Crack on”: a qualitative study of care home managers experiences and responses to system-led setbacks during the crisis of the COVID-19 Pandemic in England

**DOI:** 10.1007/s41999-023-00804-y

**Published:** 2023-06-06

**Authors:** Fiona Marshall, Adam L. Gordon, John R. F. Gladman, Simon Bishop

**Affiliations:** 1 NIHR Applied Research Collaboration-East Midlands (ARC-EM), Nottingham, UK; 2 Academic Unit of Injury, Recovery and Inflammation Sciences (IRIS), School of Medicine, University of Nottingham, Nottingham, UK; 3 Nottingham University Business School, Nottingham, UK; 4 Knowledge and Intelligence Team, Derbyshire County Council, Matlock, UK

**Keywords:** Homes for the Aged, COVID-19, Organisational Healthcare, Policy, Mētis, Systems theory

## Abstract

**Objectives:**

To explore care home managers’ experiences of systems working with various organisations, including statutory, third sector and private, during the second wave of the COVID-19 pandemic from Sept 2020 to April 2021

**Design:**

An exploratory qualitative interview study using a systems theory approach focussing on the intersections of relationship interdependencies with other organisations.

**Setting:**

Conducted remotely with care home managers and key advisors who had worked since the start of the pandemic in/with care homes for older people across the East Midlands, UK.

**Participants:**

8 care home managers and 2 end-of-life advisors who participated during the second wave of the pandemic from Sept 2020. A total of 18 care home managers participated in the wider study from April 2020 to April 2021

**Results:**

Four organisational relationship interdependencies were identified: *care practices, resources governance* and *wise working.* Managers identified changes in their care practices as a shift towards the normalisation of care, with an emphasis on navigating pandemic restrictions to fit the context. Resources such as staffing, clinical reviews, pharmaceutical and equipment supplies were challenged, leading to a sense of precarity and tension. National polices and local guidance were fragmented, complex and disconnected from the reality of managing a care home. As a response a highly pragmatic reflexive style of management was identified which encompassed the use of mastery to navigate and in some cases circumvent official systems and mandates. Managers’ experience of persistent and multiple setbacks were viewed as negative and confirmed their views that care homes as a sector ere marginalised by policy makers and statutory bodies.

**Conclusions:**

Interactions with various organisations shaped the ways in which care home managers responded to and sought to maximise residents and staff well-being. Some relationships dissolved over time, such as when local business and schools returned to normal obligations. Other newly formed relationships became more robust including those with other care home managers, families, and hospices. Significantly, most managers viewed their relationship with local authority and national statutory bodies as detrimental to effective working, leading to a sense of increased mistrust and ambiguity. Respect, recognition and meaningful collaboration with the care home sector should underpin any future attempts to introduce practice change in the sector.

## Key summary points

**Aim:**

To identify care home manager’s experiences of working within and across organisational and regulatory boundaries of practice during the second wave of the COVID-19 pandemic.

**Findings:**

Managers continued to experience challenges to resources which were predominantly system-led setbacks across the care home sector and external organisations. Managers deployed highly pragmatic and reflexive practices to ensure the safety and well-being of residents and staff.

**Message:**

It is essential that key lessons, including expertise, respect, recognition and meaningful collaboration with the care home sectors are embedded across the statutory and regulatory organisations to maximise and build on previous gains for effective future workings.

## Introduction

The COVID-19 pandemic, which started in February 2020, had devastating effects on care homes and the older people that live in them. Internationally, 41% of COVID-19 deaths during the first year of the pandemic (until Feb 2021) occurred in care homes [1]. This high mortality, and the associated systemic responses to the pandemic, have had lasting effects on the sector. Two years after the start of the pandemic, care homes were struggling with staff retention and recruitment and precarity of business models, leading to reduced bed capacity and knock on effects on the performance of the health and social care system more widely [2].

We previously reported on how UK care homes navigated the support provided during the first wave of the COVID-19 pandemic, which lasted from February to May 2020 [3]. We found that care home managers perceived the central government response to be absent or incomplete, slow, poorly co-ordinated, and insufficiently cognisant of the daily reality of care home working. Our data identified that many local primary care, social care and community care services, charged with responsibilities for the delivery of care and support in care homes, retracted from care homes. This retraction resulted in care home staff having to work at very edge of their professional boundaries as they worked towards maintaining the dignity and well-being of their residents [3]. Regulatory inspections ceased.

Our earlier data identified that many care home managers relied upon international evidence of pandemic responses, notably from Italy and China, as the pandemic spread across Europe. Drawing on international media accounts, many mangers sought to lockdown by early March 2020, limited staff movements in their homes and communities (with some choosing to live on site) and sought to update their knowledge of infection control in the context of the available evidence about transmission routes [3]. Care home managers were, at times days, or weeks, ahead of UK national policy decisions, drawing on international sources of information to guide their decision-making. They had to work around, or despite, national policies and statutory support. For example, statutory arrangements to provide Personal Protective Equipment for staff that was pledged but not forthcoming. They did this by finding advice and practical support through a combination of informal and formal supports within their local communities. This included from individual volunteers, schools, community groups and hospices. We argued that the inadequate nature of prior relationships between the care homes and the statutory sector rendered them particularly vulnerable to the challenges of the pandemic [3].

The second wave of the pandemic in the UK lasted from Sept 2020 to April 2021. In the period between the first and second waves much had changed. Guidance from the Department of Health and Social Care on managing COVID-19 in care homes was first issued in April 2020, and underwent 5 iterations by August 2020, and a further 9 iterations by April 2021 [4]. These included changes around COVID-19 symptomatology, quarantine and isolation policies, arrangements for discharges from hospitals to care homes, visits by family members, guidance on testing for COVID-19, how care home providers could access financial support and the introduction, in December 2020, of a statutory inspection regimen to establish “designated settings” which were safe to manage residents with COVID-19. A care home testing programme, including asymptomatic testing of staff and residents, where tests were sent off for laboratory analysis by Polymerase Chain Reaction (PCR), was rolled out nationally in June 2020. Guidance for members of the public visiting care homes was produced in July 2020. Additional National Health Service support, including the requirement for all care homes to have a named health professional as their primary contact, was mandated in October 2020. Lateral flow tests were piloted for visitors in December 2020 and rolled out nationally in March 2021 to replace PCR testing [5, 6]. The first national lockdown ended in June 2020. There were regional, tiered lockdowns in October 2020, a second full lockdown between November and December 2020, and a third full lockdown between January and March 2021.

Thus, between the first and second waves, and during wave 2, of the COVID-19 pandemic in the UK, care homes went from a paucity to a plethora of guidance, were subject to substantial legislative augmentations, had increased contact with healthcare staff and regulators introduced by mandate, and had markedly increased access to diagnostic technologies which they were obliged to use. The on–off nature of the lockdowns also affected the voluntary and informal community supports that we found care home managers relied upon so substantively during wave 1 had dissipated.

We worked with care home managers during wave 2 to consider how things changed during the second stage of the crisis and how they adapted to the different challenges to continuing to provide care. We aimed not only to be able to inform future pandemic preparedness, but also to provide insight into the overall resilience of the care home sector to future challenges unrelated to infectious outbreaks.

## Method

To compare experiences of care home managers during wave 2 of the pandemic with those during wave 1, the methods for the current study build closely on the research previously reported [3]. Specifically, the research continued with the ethnographic organisational systems approach, which identifies care homes as part of complex systems, embedded within structured networks of relations with other health and social care actors, and potentially affected by their actions in both anticipated and unanticipated ways. Rather than seeking to provide an objective system map from an etic perspective, our study attempts to develop an understanding of the system point of view of care home managers. In facing the large challenges and practicalities of running the care homes during multiple waves of the pandemic, we identify care home managers as well placed to provide expert insight into the workings of the health and care system, and how the processes, structures and relationships changed over a relatively short period of time.

### Data collection

The primary data collection method for the study involved qualitative interviews with care home managers. Data collection for the study took place between September 2020 and January 2021, a period during which Covid 19 cases were rising rapidly in the UK and covering the second national lockdown [7]. During this period, eight registered care home managers were recruited from within the East Midlands of England. All were from homes registered for care of older people. Each manager had been employed at the home for at least 6 months prior to the interview. Additionally, we secured interviews with a hospice educator and an end-of-life specialist GP working closely with several care homes. Participants were identified through existing care home networks, established by the research team through previous work conducted in and with the sector. We used these as a starting point for a chain referential approach to recruitment, asking members who had previously participated in research to help us identify care home managers who had not been engaged in research before. We supplemented this by asking professional care and research networks to publicise the study using social media and electronic mailing lists. We chose to focus on managers new to research because many care home managers that we had routinely interacted with in the past through our research had engaged with online support forums alongside senior clinicians and academics [8]. We considered that such experiences were likely to be atypical and we wished to explore broader accounts of how care homes responded to the crisis. Further, we wished to reduce the research burden on the managers involved in our previously reported study. Most care homes were geographically located in semi-urban or rural areas and ranged between 12 and 94 bedded units. Consent was obtained by email and took place remotely using either telephone or videoconferencing software. Each interview was taped and transcribed verbatim by a qualified transcriber, lasting 35 min on average.

### Data analysis

The interviews with care home managers and subsequent data analysis were informed by the system interdependencies identified in the first study [3]. System interdependencies capture the relationships between elements with a system, and how the actions of one element of a system impact upon and shape the subsequent (re)actions of other elements [9]]. In this case, the key interdependencies of interest were those between the care homes and other actors within the wider health and social care system, including primary and secondary health care organisations, commissioners, central government and the wider public. More specifically, our previous study identified three areas of inter-dependency with a strong bearing on care managers work. Namely these were interdependencies of care processes and practices (the everyday actions of providing care for residents), resources (the availability of people, materials and technologies required to continue care) and governance (the systems of oversight and control). In the first wave of the pandemic, care home managers reported that each of these had become severe challenges, but also that the way that these challenges were manifest was strongly shaped by actions by other actors within the heath and care system [10]. These interdependencies were therefore used as the basis for the topic guide for the current study [3].

The three previously identified interdependencies provided the starting point for thematic analysis of the data. We refined and further developed the fit between sub-themes and our overall understanding of the organisational interdependencies iteratively as we proceeded through coding [11]. Through this process, we identified that while much of the participants experience could be related to either care practices, resources or governance, a further key theme of “wise working” was required to capture the increasing mastery of crises management by the care home managers, including the ways in which they adapted and dealt with various challenges as the pandemic continued. The coding of transcripts was also informed by an awareness of the (changing) official guidance and other sources of information (international, clinical, and local) as identified in the individual accounts and official (media and online) sources. These were used to help understand points of tension and place care home managers’ experience within a knowledge of the wider context. The write up of the results attempts to convey the varied experiences of all managers interviews, without reducing differences to a unified account.

For clarification, we refer to the notion of “wise working” as derived from the work of Mary Dixon-Woods which is underscored by the theory of Mētis [12]. This is a theoretical approach which explains the practice of managers as they sought to navigate and adapt to the ongoing crisis as a means to promote optimal care for their residents and staff. Typically, mētis can refer to the highly heterogenous context of the working which demand pragmatism within the personal, collective and wider boundaries of professional practice. Embedded knowledge and expertise held by the sector leans towards predominantly learning and practice which is acquired through verbal exchange, demonstration and practical experience. This is often tacit knowledge and expertise, reliant upon the working shared understandings and knowledge sharing within the care homes setting and beyond. Mētis, in the context of healthcare, can be used to describe complex and flexible ways of working during a crisis, by use of circumvention and responsiveness to perceived (unmet) needs [12]. Scott [13], identified the triggers which tend towards the deployment of mētis include: 1. Crisis often characterised as ambiguous and changing; 2. Bureaucratic processes, often based on algorithms, which are not context specific; 3. Powerful organisations demanding outcomes which cannot be met by less powerful groups and 4. Lack of understanding or resources to complete the job, by adherence to prescribed routes, such as adherence to regulations. The deployment of mētis tends towards assured dissent, cunning, taking back of power from the most powerful and notably use of ongoing improvisation drawing on professional practice and group knowledge. Within healthcare the invocation of mētis is to avoid harm and is not a form of negative dissent on the part of the care home managers. It can be considered as a careful collective response within a community of practice, such as the care home sectors, to achieve the most ethical response during times of intense crisis as experienced during the pandemic.

## Results

Leading on from the above, our results are presented in four sections, namely care processes and practices; resources; governance; and wise working. These issues were often inter-connected, but we present them individually for clarity of explanation and understanding.

### Care processes and practices

As previously identified [3], the work of care homes is strongly affected by their peripheral position as care pathway providers, compared to NHS services. At local levels, care homes were integral to the continuance of care transitions from NHS care yet were often, dependent on the activities of other health and care organisations. Over the waves of the pandemic, the care processes and practices of the care homes could be seen to change. Many of these changes were as a result of the increased knowledge of the COVID-19 virus including testing and vaccinations. However, changes in systems resulted in concurrent setbacks as care homes struggled to keep pace with system changes.

A key issue was that care homes had to both deal with the wider health and care system returning to more ‘normal’ work, alongside COVID. More broadly, the entire system returned to normal in a fragmented and inconsistent manner, which disrupted the interfaces between care homes and external organisations and individuals with which they interface. As the wider system became busier and any slack created to deal with COVID was taken up, care home managers identified increased and repeated delays, for example, in receiving medications and obtaining specialist reviews. This required managers to their own time to chase around for a response and led to anxiety over the ability to meet the needs of residents in a timely manner. This was also perceived as evidence of the lack of prioritisation and consideration for care homes within the wider health and social care system.

As identified during the first wave [3] individual ties with healthcare professionals were seen as valuable, but most participants were concerned that their relations with the wider healthcare system continued to deteriorate. The extreme nature of the ongoing challenges within care homes exacerbated a sense of “them and us”, with care homes managers perceiving that they were outside the system.

*No, the district nurses basically because we’re a dual-registered home, so we’re nursing and residential, anybody that normally would have the input of a district nurse on the residential side, they basically came and said; “you’ve got nurses here, you need to do all the catheters, you can do all the dressings and what not. We will pay you for it but you’ve got to do it”. There’s no consideration as to we’re all struggling to find nurses. There is absolutely no consideration of the fact that we have people who already pay their fees and have their own needs, that’s why they’re classed as nursing band. And then you get them [district nurses] just shoving them on you.* *CH12, November 2020.*

The position of care homes in relation to the NHS and local authorities was also influenced by the trajectory of the pandemic and outbreaks within homes. In particular, the relatively low number of cases during the summer of 2020 followed by the rapid progression of the second wave, shaped the changing workload of care homes and the support they received. Many managers interpreted the lack of support in light of this and expressed their despair and anger towards the statutory services for not developing improved support systems during the summer lull. This applied to the NHS, local public health, and social care services.

*The clinical commissioning group was invisible, when you tried to contact them, you know … I have to say that at the time trying to contact Public Health, trying to get support was really difficult. Infection Prevention and Control were giving conflicting advice to Public Health England. And in the end, you just … to be honest I just thought this is a waste of time, we know more ourselves. And just by phoning them was just making us angry.*

CH17 December 2020

Whilst a plethora of governmental guidance specific to the sector had been produced by the winter of 2020, care home managers felt this was often disconnected from support and advice at more local levels. Relationships with statutory organisations were seen as compulsory, during the pandemic, often primarily focussed on collating data about, or monitoring activities within, care homes for those in regional and national government. Duplication of data reporting was a persistent problem and increased both workload and frustration.

Multiple iterations of guidance, and a tendency to release this late on Fridays, led to care home staff having to work through the weekend to accommodate new guidance, often without support or clarification from statutory services, whose staff were on leave until the following Monday. Paradoxically, despite the increased focus on care homes, managers felt more isolated than during the first pandemic wave—having to work through multiple information demands or mandatory guidelines without support.

*And you know we will get a weekly letter from them, [adult social care] it’s a bit hit and miss whether you receive it. And I find it’s always late on a Friday and I personally don’t like it when they end it with you know, ‘Thank you for everything that you’re doing’. And I think we’re not doing it for you.*

CH18, December 2020

*…. some home managers are more technicallysavvy than me(laughs), some of the other managers were very technically-savvy, so they would actually post the relevant stuff. But the area manager as well would always contact us, even at the weekends and say have you seen the latest guidance, you need to implement that on Monday. I think you need almost headlines, bullet points and then if you need to explore that further this is where you find it. Because very often what they’re posting isn’t relevant…*

CH12, November 2020

Whilst the overall disruption to society as a whole due to the pandemic could explain some of the experiences of care home managers it is notable that General Practitioners, themselves hard hit by the pandemic, continued to provide in person consultations with residents. This included end-of-life care and was highly valued by the care home managers as they sought to extend their knowledge to include complex care because of the diverse symptoms of the virus. At times this included highly specialised end-of-life care within settings normally associated with hospice or hospital.

*Our GPs now do ward rounds every Tuesday. They don’t come in the building as much as they did pre-Covid, they would probably come in most days, they would always come in if we phone them. Obviously now they tend to try and do video consultations or whatever…*

CH13 October 2020

Relationships with residents’ families remained important but were often conducted remotely, over the phone or using videoconferencing due to lockdown regulations. Family members were missed by the care home staff, since previously they had provided regular support to the activities and routines of the home.

*I’ve got great admiration for all the relatives, it breaks my heart that they’re not coming in because again, because we’re a small home, people would just come in and it would just be you know, every relative would speak to all of the residents. And they’d know them by name, and you know, it was all homely.*

*CH18, December 2020*

Although ties with most families remained robust, the nature of these ties had changed from close co-productive relationships to more distant and formalised in time and place. Communications, previously often face-to-face were replaced by remote discussions and the need for care home staff to explain and build consensus about care decisions for their loved ones. This was often emotionally difficult and time consuming for staff to manage. Staff disliked the nature of communications between windows, the emotional impact on residents and the lack of spontaneity.

### Resources

In the first wave of the pandemic, care home managers were found to have been ‘left out’ of systems for securing key resources [3]. During the second wave, care homes had largely secured access to PPE, and this became less of an issue. However, other resource issues emerged, related to the changing guidance and bureaucratic processes surrounding the pandemic.

A first emergent resource issue was that most homes experienced difficulties within the processes surrounding COVID-19 testing. This was poorly communicated and there were delays in delivery and collection of tests, which sometimes mean tests were invalidated because they were not analysed within the required timeframe. Delayed results contributed to uncertainty about management of staff and residents, which depended upon timely test results. Managers perceived the service as unreliable and this sometimes-encouraged staff to obtain PCR tests outside of the care home system.

The loss of trust established through unreliable PCR testing was compounded through inconsistent messaging accompanying the introduction of lateral flow tests. Care home managers felt they were often the last to know about changes that affected them.

*“the latest one with the lateral flow devices you know, people are angry that as care staff we can’t send them home with that to do at home. And it wasn’t until [a medic] put on [the shared app] the other day to say actually we can because they were passed, I think, in December. But the Department of Health and Social Care has not bothered to tell any of us. You know and I think why do you do that? Because is it that we are not trusted but you can trust NHS staff, teachers, lots of other people but you cannot trust us?”*

CH18, January 2021.

A second resource issue was that managers reported increasing difficulties retaining staff following the emotional and psychological insults of the first wave, and the ongoing stressors associated with working in the sector. Reasons for leaving were included mental and physical exhaustion, poor health (including due to COVID), family pressure to leave, and joining the NHS which offered better terms of employment. Despite government advice not to employ agency staff, several homes had no option but to resort to the regular employment of agency staff to maintain day-to-day care.

*… before we actually got Covid in this home, we had some staff that went off, some pregnant that had to go home, so we were short-staffed, so I did actually go down and work on the floor for about … between the middle of March and probably the beginning of June.*

CH14 December 2021

*So, some of our staff found it really difficult to move on. They were stuck in this ‘we’ve had Covid you know’, We did have some staff who were frightened of Covid, frightened to be here. And basically, avoiding being here.*

CH15 December 2021

As normal life resumed following the first lockdown, managers witnessed a breakdown in the early support that they had experienced from local community in accessing important resources. This included the dissolution of ties with neighbouring schools, businesses, and other organisations. Gifts and offers in kind—ranging from personal protective equipment, through food and clothing—largely stopped arriving during the second wave, although some seasonal gifts arrived close to Christmas during the December 2020 lockdown.

*that community, the community groups and relationships that we used to access, that we have relationships with, churches, tearooms, dementia cafés, those sorts of things, they all closed.*

CH15 November 2020

*I wouldn’t have known about zoning and lots of other things if it hadn’t have been for that group…none of us are nurses. [Whats App group, Care Home Care]*

CH18 December 2020

Some of the ties developed during the early pandemic did persist and become stronger. These were largely with third-sector organisations, especially in relation to end-of-life care. Ties with hospices remained strong, with a particular focus on skills and training to enable care home staff to provide high quality palliative care in the face of a COVID-19 outbreak.

*…particularly over the Easter weekend [2020], we were running short of Morphine and Midazolam. So, guidance was put together on sort of alternatives that staff might be able to use to control symptoms at end of life. So again, working in partnership with [local community health service] and rolling that training out to care homes. I’ve been involved with I suppose the Government push, work came very late in the day really on infection prevention and control for care homes.*

Hospice Coach to care homes, July 2020

### Governance

Over the course of the pandemic, the systems of governance changed rapidly. Managers reported irritation about the increased regulatory and inspection regimes introduced during the latter part of 2020, when they perceived the regulators to be lacking in competence. Overall, the information and regulation demonstrated a lack of understanding of the care homes and the ongoing care demands.

*Second time around, suddenly there’s all this reporting and there’s auditing and there’s you must do this, and you must do that, and the guidance changes every 30 seconds. It comes out on a Friday night, lastminute.com, they expect us to implement it the next day and so there’s a sense of where were you when we really needed you? Now we know how to do this, why are you telling us off?*

CH16 November 2020

Managers often felt compromised by the mismatch between high level press releases from government ministers and officials, which some families held them accountable to, and the support available to operationalise these on the ground. This was most marked around the prioritisation of residents for immunisations, which was announced in the press over the Christmas holiday period, with emphasis in the press from government spokespeople that care homes would soon be able to reopen. This led to pressure from families to renew contact with residents, whilst the reality was that vaccine roll-out was slower on the ground. This led to confusion among families and staff. Managers faced similar issues as they adhered to isolation and quarantine guidance, grappled with complex testing regimes and worked to put mandatory protections in place to enable visiting, even as they found themselves vilified in the national media for not doing enough, and for being too slow. All the managers we spoke to deeply regretted the continued visiting restrictions and were sympathetic with the residents and families.

*You know, on one hand the Government’s saying to everybody work from home, stay at home and then they’re saying but go and hug a granny in a care home…*

CH17, December 2020

### Wise working

In the first wave of the pandemic, it was found that care home managers adopted a tactical management approach, using a command-and-control approach within their homes to quick decisions in the face of severe resource shortage, high mortality and limited statutory guidance and support [3]. By the second wave, there was a resumption of a more flattened hierarchy within the homes but with managers needed to become increasingly reflexive and strategic in their actions. Many managers described their actions as a series of considered responses to regularly shifting and ambiguous demands placed upon them.

Whilst care home managers sought to (re)balance the needs posed by management and care delivery, they found themselves frequently unable to focus on care delivery due to the continued need to respond to changing environments and resources. Some homes encountered their first COVID-19 cases in the homes during the second wave, some had repeated outbreaks, whilst others managed to avoid any infections. All homes were, though, affected by scarcity of staff, which led to a reduction in bed numbers and associated anxieties of future viability of their homes and the sector. Managers had to navigate both familiar and new demands on their service. This was highly pragmatic, with a “making do” attitude combined with a more reflective and strategic approach. It was clear that the management of care homes was evolving at different rates with highly heterogenous approaches, despite the external factors such as homogeneous mandated regulation.

Managers described a new mastery of “wise working”, referring to the deliberate use of strategies which bridge the gaps between hierarchical, governmental imposed regulation to be adapted for use in the context of the care homes. The types of strategies described were creative, often included intelligent cunning, and collectively approved of by the care home staff. These included:

- Deliberate deflection to avoid protracted conversations with agencies, delaying and diversion of attention by the managers. This enabled them to get on with their jobs.

*I think when a system fails significantly because everything’s gone horribly wrong in the world, they [CQC] could have been more prescriptive. They’re saying you know; we need this but we’re not going to tell you what this is or what it looks like, we’re only going to tell you if you get it wrong. And actually, I’ve had more help from my peers in the community than I have from them… I’m telling staff that they have to sign to say that they’re well to work, etc, etc, CQC won’t do that. Seems like there’s a bit of a divide and politically I can understand the reasoning, but it doesn’t make it easy for me to explain on the floor. You just don’t know where you are from one minute to the next and that’s where that complacency kicks in of sod it. I have to line my own ducks up in a row [sort this out] in my own way.*

CH16, December 2021

- Bridging impractical guidance by invoking actions based on mētis so that the guidance appeared to be working but making it work by circumvention and redesign:

*We have to learn to live with this [Covid-19] so we have jiggled the guidance.*

CH17, November 2020

*we’ve got syringe drivers here anyway and the nurses are okay with doing them. And if it’s on the residential side, we get the authorisations. The district nurses are meant to come in and do them but obviously our nurses are compassionate and will actually give the medications that are needed.*

CH11, October 2020

- Collectively seeking power by joining together, seeking out policy makers, and going to the media to highlight challenges in practice.

*There was definitely times that I was definitely out of control. Very, very irritated by the lack of guidance, the lack of understanding and the lack of clarity. And rather than just moan about it, I did try to work nationally and locally.*

CH17, December 2020

- Drawing on multiple sources of expertise and experience, often from all grades of staff, to arrive at consensus towards decision-making (collective agreement part of mētis working).

*So, then it was like well I’ve been told to wait, so you know I’m going to wait, I’m doing as I’m told. Nothing comes through, nothing comes through [from central vaccination programme]. There’s a couple of care homes where they’ve had it but you know, nothing for us. So then eventually we did get the vaccination but through our local surgery. All my staff were vaccinated in one session and all my residents the next day [by the local GP surgery]*

CH18, January 2021

Key to resilience, was the strong support that care home managers provided to each other. This was often from colleagues that they’d regarded to be competitors for business pre-pandemic. This included sharing resources urgently, knowledge sharing and generally giving emotional support:

*…in reality as a business financially we are in competition, we are, that’s the reality. But at the same time, you know, I know a lot of other managers, I know a lot of the other homes you know, and I feel if we support each other and if they get better, then we get better.*

CH15, December 2020

*Yes, we are a community and we’re trying to help each other. Whereas before this we were all fighting for, I’ll have that one, no I want that one and we were fighting over the residents.*

CH12 November 2020

## Discussion

Our main findings are that during the second wave of the pandemic many of the ties that managers had relied on during the first wave changed. These dissipated (such as with suppliers), returned to pre-pandemic arrangements (such as social care and primary care), or evolved into more robust ties (such as primary care, other care homes, statutory regulatory bodies). Ties newly formed during the first wave were precarious and tended to be short-lived, as organisations such as local schools and businesses returned to their regular commitments. The value of the remaining ties, from the perspective of the managers, were variable. Some established ties were considered increasingly negative and disconnected from the business of care home work, such as those with regulatory bodies and local public health and social service departments, which were seen as generated bureaucracy and demand without cognisance of the lived reality in care homes. Other ties, including with families, hospices and primary care were highly valued, even though many had to be reconfigured due to COVID-19.

The more local and engaged organisations were with the care homes during the pandemic, the more aligned they became to supporting the care home managers as they sought to (re)build ties and sustained connections. Our findings indicated that there were persistent inequalities in the perceptions of the role of care homes as responsive partners in the pandemic policy and practice. Underpinning this may have been the secondary nature of community care as perceived by the NHS and statutory bodies tasked with supporting the care homes during the pandemic. It is clear that the persistent absence of genuine partnership workings at local and national levels exposed the priorities and pressures which these organisations faced and the inequitable distribution of resources and governance. These findings are not unique to England and have been highlighted across Europe and the USA [14, 15] 

The most fragmented and consistent challenges identified were those ties with geographically remote statutory organisations, as they introduced mandatory measures during the second wave, which generated new difficulties for care home managers. There was an assumption that more geographically isolated care homes were able to source sufficient workforce members and were able to access digital communications; this was often not the case. Smaller, secure units expressed concerns that contact by statutory bodies was scant and staff had little opportunities to take annual leave or work shorter hours. Rapidly changing, sometimes contradictory, vague advice from different national and regional government agencies, left care home managers and their teams struggling to keep up and undermined their trust in the guidance they were mandated to follow. Lack of consultation, for example around the way in which community health services supported care homes and around the resumption of regulatory inspections, left managers feeling compromised, exposed and “outside the system”. Consequently, multiple system setbacks were encountered by care home managers. These fostered a sense of mistrust and lack of understanding of the needs of the sector. Managers and staff addressed these setbacks using mētis and increased their managerial skill sets to help mitigate the negative impacts of the pandemic.

The care home sector in the UK comprises a disparate mix of small, medium and large providers with business models, which range from third-sector philanthropic providers to large corporations with shareholders. None of the managers we spoke to expected to have played a direct role in designing national responses to the pandemic, but they recognised little policy understanding of how care homes work day-to-day in the tasks they were asked to carry out or the protocols they were asked to adopt. This has been described elsewhere, where COVID testing procedures, regarded as straightforward by those writing guidelines, generated substantial work for care home staff as they worked at scale to take account of the needs of staff and residents [6, 13]. Later in the pandemic, the mandatory vaccination of care home staff, subsequently abandoned due to consequences unforeseen by government but outlined in advance by representatives of the care home sector [16], was a further example of the mismatch between central edict and the reality on the ground. In countries where care homes are organised in a more centralised way this clash was less self-evident—in Germany and Denmark, for example, care homes were subject to more immediate and specific control from government agencies, with better co-ordination as a consequence [14]. In the UK, by contrast, there was no obvious direct line of command nor, though, were care homes given agency to learn and adopt practices from other countries in the way they had during the first wave.

In our previous research care home staff told us they felt that they and their residents were abandoned during the early stages of the pandemic [3]. In this second wave, they told us that they were overwhelmed by mandates that were profoundly difficult to implement on the ground, such as invoking the guidance on family visits. Specific challenges were faced by residents who could not see their loved ones as often as usual with staff, often working with lowered staff numbers, trying to meet the well-being needs of individual residents and also ensure the safety of the home as a whole. These issues were seen in other countries and the UK did not rapidly learn lessons from other countries that reopened visiting, safely, early in the pandemic [17, 18]. The unifying theme across both waves of the pandemic was that the expertise of managers and their staff was under-recognised and underutilised by those in central government. Research conducted pre-pandemic illustrated the pivotal role played by care home staff in effective service design, implementation, and improvement [19, 20]. These depend on engaging staff to understand the organisational attributes of each care home [15], understanding how the priorities of staff and residents subtend improvement initiatives [21, 22], and enabling them to use their expertise of their own setting and clients to develop care home-specific solutions [23–25]. Increased contact with General Practitioners during the second wave was universally regarded by respondents as positive. This corresponded with an initiative to have a named NHS lead clinicians for every care home in the country—based upon a pre-pandemic initiative to build relationships with and around care homes, enabling them to participate in more integrated care delivery [10, 26, 27], an approach quite different from the management by edict evidenced elsewhere. Governmental steps taken later in the pandemic, such as the appointment of a national lead nurse for social care drawn from the sector [28] are focussed around engaging the support of, and harnessing the expertise, within care homes.

Limitations of this study include the limited timespan, which covered only the last part of 2020, and could not continue beyond January 2021. However, as described earlier in this article, this was an important time of substantial change for care homes. Conducting focussed research during this time, and drawing comparisons with earlier research, has enabled generalised lessons about which consistent approaches taken by managers and staff during this time enabled effective care delivery. The geographical frame of reference was also narrow, yet the findings have face validity when considered as a representation of the situation across the UK when compared with other studies, described above, of how care homes responded to central mandates during this period. The findings are not generalisable to settings outside of the UK but do provide an important case study when considering, more generally, how organisational structure must be taken into account when introducing rapid cross-sectoral change. We did engage with care home managers who had previously never taken part in research and found their views differed from the previous care home managers recruited for the earlier part of the study. It is important to enable the participation of lesser heard care home managers to elicit their views and experiences in addition to the more research active sites. Follow-up work would be of value to establish how the care home managers have adapted to the changes post-pandemic which include the ongoing impact of stresses within the wider health and social care systems.

The lessons for the future, from both this and our earlier study, relate to the need to recognise, respect and engage the expertise embodied by care home staff. National guidelines must be designed with sufficient flexibility to enable local adaptation, and the importance of relationships with individuals and organisations within the localities of care homes recognised. Consultation and co-design are central to such adaptation, with an emphasis on mutual respect and trust. This should include care home leaders and front-line staff and—as the well-rehearsed issues with visiting highlight—finding ways to hear, respond to and incorporate the views of residents and their families is key. With the advent of newly formed local NHS, Social care and public health bodies coming together as Integrated Care Systems in England [29], it is hoped that these will proactively seek to collaborate and draw on the expertise held within the care home sectors. Care home, NHS and third sector staff within localities are well-placed to be able to hear and respond to the needs of residents and families. Advocacy groups, such as Healthwatch England, The Carers Association and others can hold key roles in providing oversight for statutory and provider organisations. When such needs are heard and recognised, the worst excesses witnessed in the pandemic can be avoided, and optimal, person-centred care advanced.

## Data Availability

The datasets generated and/or analyzed during the current study are not publicly available due the sensitive nature of the data but are available from the corresponding author on reasonable request.

## Declarations

### Patient and public involvement

Care home mangers were involved in the design of the study. It was not possible to consult with residents or family members during the pandemic.

### Provenance and peer review

Not commissioned; externally reviewed.
